# Validation of the Dutch translation of the quality of recovery-15 scale

**DOI:** 10.1186/s12871-022-01784-5

**Published:** 2022-08-01

**Authors:** Johannes C. N. de Vlieger, Willem H. Luiting, Jessica Lockyer, Peter Meyer, Joke Fleer, Robbert Sanderman, J. K. Götz Wietasch

**Affiliations:** 1 Department of Anesthesiology, University Medical Centre Groningen, Hanzeplein 1, 9937 GZ Groningen, the Netherlands; 2 Department of Anesthesiology, Leiden University Medical Centre, Albinusdreef 2, 2333 ZA Leiden, the Netherlands; 3 Department of Health Psychology, University Medical Centre Groningen, Hanzeplein 1, 9937 GZ Groningen, the Netherlands

**Keywords:** Outcome assessment, Patient-reported outcome measure, Postoperative period, Quality of health care, Questionnaire

## Abstract

**Background:**

The 15-item Quality of Recovery-15 (QoR-15) scale is strongly recommended as a standard patient-reported outcome measure assessing the quality of recovery after surgery and anesthesia in the postoperative period. This study aimed to validate the Dutch translation of the questionnaire (QoR-15NL).

**Materials and methods:**

An observational, prospective, single-centre cohort study was conducted. Patients who underwent surgery under general anesthesia completed the QoR-15NL (preoperatively (t1) and twice postoperatively (t2 and t3)) and a visual analogue scale (VAS) for general recovery at t2. A psychometric evaluation was performed to assess the QoR-15NL’s validity, reliability, responsiveness, reproducibility and feasibility.

**Results:**

Two hundred and eleven patients agreed to participate (recruitment rate 94%), and 165 patients were included (completion rate 78%). The QoR-15NL score correlated with the VAS for general recovery (rs = 0.59). Construct validity was further demonstrated by confirmation of expected negative associations between the QoR-15NL and duration of surgery (rs = -0.25), duration of Post Anesthesia Care Unit stay (rs = -0.31), and duration of hospital stay (rs = -0.27). The QoR-15NL score decreased significantly according to the extent of surgery. Cronbach’s alpha was 0.87, split-half reliability was 0.8, and the test–retest intra-class coefficient was 0.93. No significant floor- or ceiling effect was observed.

**Conclusion:**

The QoR-15NL scale is a valid, easy-to-use, and reliable outcome assessment tool with high responsiveness for patient-reported quality of recovery after surgery and general anesthesia in the Dutch-speaking population. The QoR-15NL’s measurement properties are comparable to the original questionnaire and other translated versions.

**Trial registration:**

not applicable.

**Supplementary Information:**

The online version contains supplementary material available at 10.1186/s12871-022-01784-5.

## Background

Recovery after surgery and anesthesia is a complex process dependent on patient, surgical, and anesthetic characteristics, as well as the presence of any adverse sequelae [1]. In the past, commonly reported outcome measures were recovery times and function, avoidance of common adverse effects (i.e. pain, nausea and vomiting) and healthcare resource utilisation (i.e. duration of intensive care unit and hospital stay) [2]. Although these parameters are essential and should be measured, they mostly ignore the quality of recovery (QoR) from the patient’s perspective [1].

QoR scales have been developed for the immediate postoperative period to provide a quantitative measure of overall health status after surgery and anesthesia. One of the strengths of these scales is the integration of a more complete range of patient experiences after surgery to avoid undue emphasis on one, or some over others (e.g. opioid pain reduction at the expense of nausea or delirium). The 40-item QoR scale, 15-item QoR scale and 9-item QoR score have been studied most extensively [2].

The multidimensional 15-item QoR (QoR-15) scale was initially developed in English and translated and validated in several European and Asian countries [1, 3–8]. The questionnaire assesses both physical and mental well-being. The 15 items incorporate five dimensions of health: physical comfort (*n* = 5), physical independence (*n* = 2), pain (*n* = 2), emotional state (*n* = 4) and psychological support (*n* = 2). All items are scored by an 11-point numerical rating scale. Consequently, after summing up all items, the total score ranges from 0 to 150 (ideal health status) [1, 7].

The QoR-15 scale is a valid, reliable and easy-to-use patient-reported outcome measure (PROM) with high responsiveness [1, 9, 10]. Furthermore, a systematic review following the COnsensus-based Standards for the selection of health Measurement INstruments (COSMIN) checklist showed that the QoR-15 fulfilled the requirements for outcome measurement instruments in clinical trials [9, 11]. Currently, the QoR-15 is strongly recommended as a standard outcome measure of QoR in clinical research relating to surgery and anesthesia [9]. Perioperative interventions that result in a change in QoR-15 score of 6 signify a clinically important improvement or deterioration [10, 12]. Furthermore, it could be a useful outcome measure for assessing the impact of healthcare delivery changes for quality assurance purposes [1]. Finally, the QoR-15 offers the opportunity for a standardised feedback measure for healthcare team members, especially anesthesiologists and surgeons, to acquire additional insights into their patient’s outcome.

Although validated in various linguistic and cultural contexts, the QoR-15 has never been translated into Dutch according to international standards for translating a questionnaire [4, 7, 13, 14]. Therefore, this study aims to validate the Dutch translation of the QoR-15 scale questionnaire (QoR-15NL). It was hypothesised that the QoR-15NL scale’s measurement properties would be satisfactory and comparable with the original and subsequently translated versions of the questionnaire.

## Materials and methods

Prior to commencement, the study was registered in the University Medical Center Groningen (UMCG) Research Register (201,900,402). The study protocol was reviewed and declared to be outside the scope of the Medical Research Involving Human Subjects Act by the Medical Ethics Review Board of the UMCG (METc 2019/331, chairperson Prof W.A. Kamps) on June 18^th^ 2019.

### Translation and cultural adaption

First, two independent translators from the University of Groningen Language Centre conducted the forward and backward translation of the original QoR-15 questionnaire (1). An expert panel consisting of two anesthesiology residents (JdV, JL), two senior anesthesiologists (PM, GW) and two experienced clinical psychologists (JF, RS) critically reviewed the resulting QoR-15NL pilot version and applied two modifications. Subsequently, cognitive interviews about the pilot version with patients who underwent various inpatient elective surgical procedures under general anesthesia were conducted approximately 24 h postoperatively at the surgical ward, using a structured interview guide. These interviews assessed the questionnaire’s instructions, recall, items, response options, format and length [15]. Interview transcripts were transcribed verbatim, coded (inductive) and analysed by two authors independently (JdV, JL) [16]. Eighteen patients were interviewed in three interview rounds until no new comments arose. After the first (*n* = 7) and second round (*n *= 5) of interviews, the expert panel modified the pilot version to address relevant patient comments. Consensus was reached about adding a short instruction and example about completing the questions, and three questions (6,9 and 10) were slightly modified. All relevant comments and modifications made during the translation and cultural adaption are summarised (see supplementary file 1). The resulting final version of the QoR-15NL is available at https://www.umcg.nl/-/medisch-wetenschappelijk-onderzoek/gaps.

### Validation study

During the validation study, an observational, prospective, single-centre cohort study was conducted at a tertiary referral centre between August 24^th^ and November 29^th^ 2020. Adult patients, who underwent various inpatient elective surgical procedures under general anesthesia, were fluent in Dutch, and available for follow-up at the hospital on the first postoperative day were eligible for inclusion. Patients were ineligible or excluded if they did not give consent, were admitted on the Intensive Care Unit (ICU) (both scheduled and unscheduled) postoperatively, were admitted on the Postoperative Anesthesia Care Unit (PACU) for the first night postoperatively; or if they had either poor Dutch comprehension, a psychiatric disturbance that precluded complete cooperation, a known history of alcohol or drug dependence, any severe pre-existing medical condition that limited objective assessment after surgery, any life-threatening postoperative complication or a postoperative delirium [1]. Eligible patients were contacted by phone one week prior to the intended surgery. Participating patients received an information letter, an informed consent form and two QoR-15NL questionnaires by mail. As per the development study, patients completed the informed consent form and the first QoR-15NL questionnaire preoperatively (t1, baseline) and the second (t2) on the first postoperative day.

Additionally, at t2, a 100 mm visual analogue scale (VAS), marked from ‘poor recovery’ to ‘excellent recovery’, for general recovery was added to assess validity [1]. Approximately 24 h postoperatively, a researcher visited participating patients on the surgical ward. Written informed consent was obtained from all patients, and both questionnaires were collected during the visit. Every second patient was asked to repeat the QoR-15NL(t3) 30 to 60 min after t2 to measure test–retest reliability. The time interval between measurements was in line with the development study, and visiting half of the patients would result in an adequate sample size for the analysis [17].

Patient demographics, pre-, intra- and postoperative data were collected from the electronic hospital information system. The following data were recorded: gender, age, American Society of Anesthesiologists (ASA) physical status score, time of admission, duration of surgery, type of surgical procedure, duration of postoperative stay, and postoperative complications within the first postoperative day. The extent of surgery was classified as minor, intermediate, or major depending on the type of surgical procedure and the expected surgical stress response [1, 4]. The type of surgery was classified according to the surgical subspecialty [1]. The duration of surgery was determined by using the surgery start and stop times from the hospital’s perioperative information system [1]. The duration of postoperative stay in the PACU and the length of postoperative admission at the hospital were calculated using the surgery stop time and discharge time to the surgical ward and from the hospital, respectively [4].

### Statistical analysis

The recommended sample size to validate a questionnaire is 10 participants per item [17, 18]. This study aimed to include 165 patients, accounting for a 10% loss to follow-up. Data are presented as mean ± standard deviation (SD), median (interquartile range (IQR)) or number (percentage) as appropriate. The recruitment rate represents the percentage of eligible patients who were contacted by phone and agreed to participate. The completion rate represents the number of patients who agreed to participate and were included in the study. Normal distribution was assessed with the Shapiro–Wilk test. Changes from baseline were compared by the paired t-test. Differences in QoR-15NL score for gender, complicated cases versus uncomplicated cases, and poor versus good recovery were compared by the unpaired t-test. Differences in the QoR-15NL score between the extent of surgery were compared by the one-way ANOVA test. Correlation coefficients were used to assess associations between variables: Pearson (r) for normally distributed and Spearman rank (rs) for non-normally distributed variables, respectively [19]. Statistical analyses were performed with SPSS Statistics version 23.0 (IBM Corp, Armonk, NY, USA). The null hypothesis was rejected if two-tailed *p* < 0.05.

### Psychometric evaluation

The psychometric evaluation of the QoR-15NL was performed similarly to the original publication and the subsequent translation and validation studies [1, 4, 7].

#### Construct validity

Construct validity was assessed using convergent- and discriminant validity. Convergent validity was determined by comparing the QoR-15NL with the VAS for general recovery, and inter-item correlations were measured [1]. Additionally, it was further tested by the hypothesis that there would be a negative association between the QoR-15NL (t2) and duration of surgery, duration of stay in the PACU, and duration of postoperative hospital stay [1]. The association between the QoR-15NL and age was also determined, although previous studies reported contradictory results regarding the degree and magnitude of this association [1, 4, 7]. Finally, it was hypothesised that the QoR-15NL score would be inversely related to the extent of endured surgery and that women would have a lower score than men; since women generally have a worse postoperative recovery [4, 20].

Discriminant validity was tested by the hypothesis that patients with complications and those who had undergone a poor postoperative recovery (defined as a VAS for general recovery of < 70 mm versus > 70 mm for a good recovery) would have a lower QoR-15NL score [1].

#### Reliability, responsiveness and reproducibility

Reliability was tested with internal consistency (Cronbach’s alpha) and split-half reliability [17, 21, 22]. Responsiveness was assessed with Cohen’s effect size and the standardised response mean (SRM) [17, 23]. Reproducibility was tested by evaluating agreement (smallest detectible change (SDC individual)) and the test–retest reliability (intraclass correlation coefficient (ICC) for agreement (two-way random effect model)) [17, 24]. Patients with a time interval of > 90 min between t2 and t3 were excluded from the test–retest analysis to assure that the remaining patients’ clinical condition was stable between measurements, which is required for a reliable test–retest analysis.

#### Clinical feasibility

Clinical feasibility was determined by the recruitment- and completion rate (see above). Finally, floor or ceiling effects were present if more than 15% of the respondents achieved the lowest or highest possible score, respectively [17]. Missing items were handled as follows: in case of one missing QoR-15NL item, the worst possible score (0) was selected. Two or more missing items resulted in an invalid QoR-15NL score and exclusion. Table 1 summarises the statistical methods used for the psychometric evaluation of the QoR-15NL scale.

**Table 1:** Summary of statistical methods used for the psychometric evaluation of the QoR-15NL scale

**Concept**	**Parameter**	**Abbreviation**	**Interpretation**	
**Construct validity**				
Correlation	Spearman's rho	rs	moderate	0.40—0.69
			strong	0.70—0.89
			very strong	0.90—1.00
**Reliability**				
Internal concistensy	Crohnbach's alpha		good	0.70—0.90
	Split-half reliability		unaccaptable	< 0.70
			fair	0.70—0.79
			good	0.80—0.89
			excellent	≥ 0.90
**Responsiveness**				
	Cohen's effect size		large	≥ 0.8
	Standardised response mean	SRM	large	≥ 0.8
**Reproducibility**				
Agreement	Individual smallest detectible change	SDC	good	SDC < minimal clinically
				important difference
Test–retest reliability	intraclass correlation coefficient	ICC	good	> 0.7
	(two-way random effect model)			
**Clinical feasibility**				
Floor effect	Percentage of patients with		present	if > 15%
	lowest possible score			
Ceiling effect	Percentage of patients with		present	if > 15%
	highest posible score			

## Results

Of the 224 eligible patients approached by phone, 211 agreed to participate (recruitment rate: 94%).

**Fig. 1:**
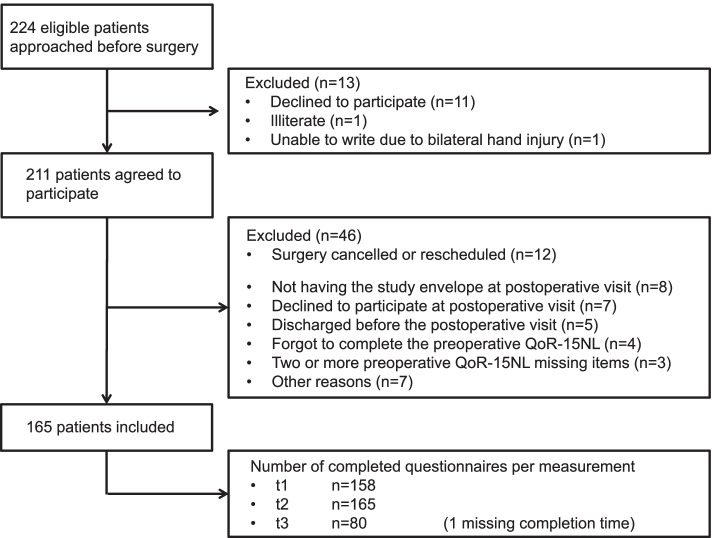
Study flow chart. Legend:QoR-15NL = Dutch Quality of Recovery-15 scale

One patient was unable to complete the postoperative QoR-15NL scale. Thirteen patients returned QoR-15NL scores with missing items: nine at t1, four at t2 and none at t3. Most patients omitted one item, but three QoR-15NL scores (all t1) were considered invalid due to the omission of two (*n* = 2) or three items (*n* = 1). After excluding 46 patients, 165 patients were included in the study (completion rate: 78%), as shown in the flow diagram (Fig. 1).

All patients underwent general anesthesia, and 22 patients received additional analgesia with an epidural catheter (*n* = 13), a peripheral nerve catheter (*n* = 4), single-shot peripheral nerve block (*n *= 4) or wound catheter (*n* = 1). Table 2 shows the demographics and clinical characteristics of the study population.

**Table 2:** Demographic and clinical characteristics of the study population (*n *= 165)

Age; years	
Mean (SD)	55 (15)
Range	19—90
Gender	
Female	100 (61)
Male	65 (39)
Preexisting medical conditions	
Respiratory	29 (18)
Cardiovascular	60 (36)
Neurological	15 (9)
Renal	12 (7)
Gastrointestinal	27 (16)
Endocrinological	24 (15)
Oncological	32 (19)
Other	66 (40)
Any medical condition	127 (77)
ASA physical status	
I	29 (18)
II	99 (60)
III	36 (21)
IV	1 (1)
Extent of surgery	
Minor	48 (29)
Intermediate	90 (55)
Major	27 (16)
Type of surgery	
Neurosurgery	42 (25)
General surgery	36 (22)
Plastic surgery	21 (13)
ENT or faciomaxillary	20 (12)
Orthopedics	15 (9)
Transplant surgery	13 (8)
Urology	10 (6)
Gynaecology	8 (5)
Duration of surgery; min (range)	135 (95–187)
PACU stay; min (range)	144 (106–208)
Hospital stay; days (range)	2 (1–4)
Postoperative complications	
Any complication	11 (7)
COPD exacerbation	1
Postoperative bleeding	2
Hypotension (vasovagal)	1
Dural leak	1
Opioid intoxication	1
Residual paralysis	1
Urinary retention	1
PONV	2
Anticoagulants during epidural analgesia	1

Results are presented as number (%) or median (interquartile range) unless otherwise stated. *ASA* American society of Anesthesiologists, *COPD* Chronic obstructive pulmonary disease, *ENT* Ear, nose, or throat, *PACU* Post-anesthesia care unit, *PONV* Postoperative nausea and vomiting

**Fig. 2:**
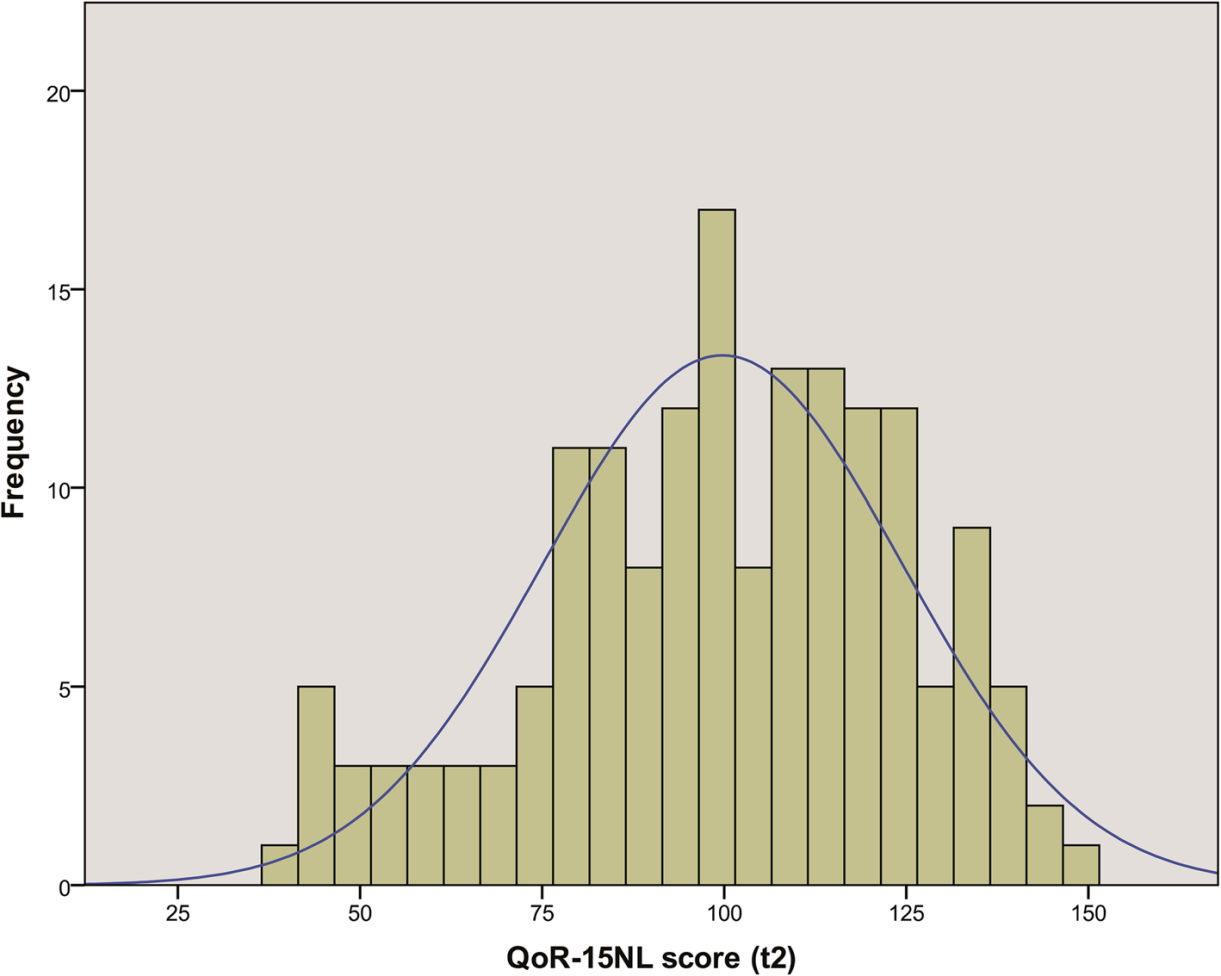
Distribution of the Dutch Quality of Recovery-15 scale (QoR-15NL) scores. Legend:Histogram, including distribution curve (blue line). The distribution was skewed to the left

The mean ± SD preoperative (t1) and postoperative (t2) QoR-15NL scores were 124 ± 18 (*n* = 158) and 100 ± 25 (*n* = 165), respectively. The mean difference between t1 and t2 was 23.5 ± 26 (*p* < 0.01). The distribution of the postoperative (t2) QoR-15NL scores was skewed to the left (skewness -0.402) and is presented in Fig. 2. Detailed data about each item of the QoR-15NL is shown in Table 3. The median (IQR) time of postoperative assessment (t2) was 21 h (IQR 18, 22) (*n* = 165), and the median interval between t2 and t3 was 56 (IQR 45, 90) (*n* = 79) minutes.

**Table 3:** Mean values, change and responsiveness of the Dutch Quality of Recovery-15 scale (QoR-15NL)

QoR-15NL item		Preoperative	Postoperative	Absolute change from baseline	Baseline change	Cohen's effect size (*n* = 158)	Standardised
		**QoR-15NL (*n* = 158)**	**QoR-15NL (*n* = 165)**	**(95% confidence interval (*n* = 158)**	**(%; *n *= 158)**		**response mean (*n* = 158)**
1	Able to breathe easy	9.5 (1.2)	8.5 (1.9)	-1.0 (-1.3; -0.7)	11	0.59	0.49
2	Been able to enjoy food	8.9 (1.9)	6.8 (3.1)	-2.1 (-2.5; -1.6)	24	0.77	0.68
3	Feeling rested	7.7 (1.9)	5.6 (2.7)	-2.1 (2.5; -1.7)	27	0.89	0.76
4	Have had a good sleep	7.5 (2.1)	5.3 (2.8)	-2.2 (2.8; -1.8)	29	0.93	0.74
5	Able to look after personal toilet and hygiene unaided	9.5 (1.4)	5.8 (3.3)	-3.7 (-4.2; -3.1)	39	1.4	1.05
6	Able to communicate with family or friends	9.7 (0.9)	8.4 (2.3)	-1.3 (-1.7; -0.9)	20	0.75	0.55
7	Getting support from hospital doctors and nurses	9.0 (1.7)	9.2 (1.4)	0.2 (-0.2; 0.5)	2	-0.07	0.06
8	Able to return to work or usual home activities	7.8 (2.9)	2.2 (2.9)	-5.6 (-6.3; -5.0)	72	1.98	1.38
9	Feeling comfortable and in control	8.1 (2.1)	6.3 (2.8)	-1.8 (-2.3; -1.3)	22	0.73	0.57
10	Having a feeling of general well-being	8.3 (1.6)	6.7 (2.5)	-1.6 (2.1; -1.3)	19	0.79	0.65
11	Moderate pain	6.6 (3.6)	5.0 (3.0)	-1.6 (-2.2; -1.0	24	0.49	0.40
12	Severe pain	8.0 (3.1)	6.9 (2.9)	-1.1 (-1.6; 0.5)	14	0.36	0.29
13	Nausea or vomiting	9.0 (2.7)	7.7 (3.2)	-1.3 (-1.9; -0.7)	14	0.42	0.33
14	Feeling worried or anxious	7.0 (2.7)	7.6 (2.8)	0.6 (0.1; 1.0)	9	-0.22	0.17
15	Feeling sad or depressed	7.8 (2.8)	8.1 (2.7)	0.3 (-0.2; 0.7)	4	-0.11	0.08
Total		124.0 (18.4)	99.8 (24.8)	-24.2 (-28.2; -20.1)	20	1.11	0.93

Mean (standard deviation) or (95% confidence interval)

### Psychometric evaluation

#### Construct validity

Convergent validity was demonstrated by the significant correlation between the QoR-15NL (t2) and the VAS for general recovery (rs = 0.59, 95% confidence interval (CI): 0.47—0.69, *p* < 0.01). The inter-item correlation matrix is shown in Table 4. Additionally, multiple hypotheses were tested. First, there was a negative correlation between the QoR-15NL and duration of surgery (rs = -0.25, 95% CI: -0.39—-0.10, *p* = 0.01), duration of stay on the PACU (rs = -0.31, 95% CI: -0.44—-0.16, *p* < 0.01), and duration of postoperative hospital stay (rs = -0.27, 95% CI: -0.41—-0.12, *p* < 0.01). The QoR-15NL also correlated with patient age (rs = 0.23, 95% CI: 0.08—0.37, *p* = 0.03). Furthermore, the postoperative QoR-15NL decreased according to the extent of surgery; patients who underwent minor surgery reported a mean score of 112 ± 20 versus 96 ± 26 and 91 ± 22 for intermediate or major surgery, respectively (*p* < 0.01). Men reported higher mean QoR-15NL scores than women: 105 ± 22 versus 96 ± 26 (*p* = 0.02). Discriminant validity was tested by two hypotheses. Patients who experienced a postoperative complication reported lower mean QoR-15NL scores than uncomplicated cases; 84 ± 21 versus 101 ± 25 (*p* = 0.04). Additionally, patients who experienced a poor recovery also reported a lower mean QoR-15NL score than patients who experienced a good recovery, 87 ± 23 versus 112 ± 19 (*p* < 0.01).

**Table 4:** Inter-item correlation matrix for the Dutch Quality of Recovery-15 scale (QoR-15NL)

**QoR-15NL item**	**Total QoR-15NL score**	**QoR-15NL item**														
	**(t2)**	1	2	3	4	5	6	7	8	9	10	11	12	13	14	15
1	**0.46**	-	**0.48**	**0.34**	**0.21**	**0.28**	**0.48**	**0.50**	0.06	**0.33**	**0.39**	**0.29**	**0.23**	**0.16**	0.09	0.13
2	**0.66**		-	**0.56**	**0.23**	**0.31**	**0.47**	**0.30**	**0.35**	**0.43**	**0.62**	**0.26**	**0.21**	**0.51**	**0.25**	**0.21**
3	**0.70**			-	**0.51**	**0.40**	**0.46**	**0.33**	**0.36**	**0.38**	**0.58**	**0.24**	**0.33**	**0.38**	**0.31**	**0.33**
4	**0.49**				-	**0.25**	**0.30**	**0.19**	**0.30**	**0.28**	**0.37**	**0.26**	**0.25**	-0.01	**0.20**	**0.20**
5	**0.68**					-	**0.58**	**0.31**	**0.48**	**0.49**	**0.51**	**0.29**	**0.29**	**0.21**	**0.33**	**0.44**
6	**0.69**						-	**0.50**	**0.25**	**0.44**	**0.62**	**0.30**	**0.33**	**0.28**	**0.37**	**0.46**
7	**0.44**							-	0.02	**0.37**	**0.39**	**0.25**	**0.17**	0.06	**0.23**	**0.32**
8	**0.53**								-	**0.37**	**0.40**	0.15	**0.17**	**0.30**	**0.26**	**0.25**
9	**0.70**									-	**0.71**	**0.30**	**0.32**	**0.32**	**0.38**	**0.33**
10	**0.82**										-	**0.41**	**0.35**	**0.41**	**0.45**	**0.44**
11	**0.52**											-	**0.59**	0.09	**0.18**	**0.23**
12	**0.54**												-	0.15	**0.24**	**0.26**
13	**0.51**													-	**0.45**	**0.38**
14	**0.59**														-	**0.72**
15	**0.61**															-

Results are presented as Spearman’s correlation coefficients, bold values indicate statistical significance (*p* < 0.05)

#### Reliability, responsiveness and reproducibility

The reliability indices of the QoR-15NL were high; Cronbach’s alpha and split-half reliability for the postoperative QoR-15NL (t2) were 0.87 and 0.8, respectively. Both responsiveness measures indicated excellent values with a Cohen effect size of 1.11 and a standardised response mean of 0.93. Compared to t2, the mean QoR-15NL score increased by 4 ± 11.6 (*n* = 80) points at t3 (*P* < 0.01). Reproducibility was considered good: the SDC was 3.6 and the ICC for test–retest reliability was 0.93 (95% CI: 0.88—0.96, *p* < 0.01) (*n* = 63).

#### Clinical feasibility

No significant floor- or ceiling effect was observed. None of the patients reported the worst possible QoR-15NL score (0), and the maximum score (150) was reported by nine (5.7%) patients (all at t1).

## Discussion

This study demonstrates the validity, reliability, and clinical feasibility of the QoR-15NL scale to measure patient-reported QoR for the Dutch-speaking population. The hypothesised satisfactory measurement properties and comparability to the original version of the questionnaire were confirmed.

In addition to translating and validating the QoR-15NL following international standards, this study has four more strengths [4, 7, 13, 14]. First, only minor cultural adaptions were necessary to adapt the QoR-15 scale for the Dutch-speaking population. Second, a clear example was added to part A’s instructions, and the reverse score for negative items was highlighted in part B to improve the scale’s comprehensibility for patients. Third, the time interval between t2 and t3 was comparable to most previous studies, making differences due to recall bias unlikely [1, 3, 4, 6–8]. Fourth, an easy-to-use method of handling missing items was introduced, which might improve the external validity and clinical feasibility of the QoR-15NL scale.

This study has limitations. As in previous studies, a single-centre study was performed, possibly limiting generalizability. Second, 46 eligible patients agreed to participate preoperatively but were not included. The leading causes of non-inclusion are shown in Fig. 1. However, it is unlikely that these non-inclusions compromised the psychometric evaluation of the QoR-15NL. For example, patients who did not bring the questionnaires to the hospital were unaware of their future QoR, making it unlikely that the QoR evaluation was subject to a non-response bias [24]. Furthermore, supposing that all seven patients who declined participation during the postoperative visit (3.3% of participating patients) could not complete the questionnaire due to poor postoperative recovery, it does not meaningfully limit the questionnaire’s clinical feasibility. Third, the test–retest analysis was performed after excluding 17 patients due to having a time interval of > 90 min between the two measurements (*n* = 16) and a missing time of completion of the questionnaire at t3 (*n* = 1). By including patients with an interval of < 90 min between measurements, the test–retest analysis was still performed with an adequate sample (*n* = 63), compared to 24 to 25 patients in most previous studies [1, 3, 4, 6, 7, 17]. The ICC was slightly lower than in the original QoR-15 study and most subsequent validation studies. However, the test–retest reliability of the QoR-15NL was still excellent [21]. Fourth, this study did not assess the minimal clinically important difference (MCID) of the QoR-15NL. An SDC lower than the MCID is a criterion for good responsiveness (the ability to discriminate meaningful clinical change from measurement error) [17]. The QoR-15NL’s SDC was 3.6, and a previous study demonstrated that the original QoR-15 had an MCID of 6 [10, 12]. Finally, no ambulatory patients were included.

This study’s findings align well with validation studies of other translated QoR-15 versions. As reported in the Korean validation study, patients reported some difficulty with the reverse score for negative items at part B during the cognitive interviews [8]. Furthermore, construct validity was confirmed by an association between the postoperative QoR-15NL score (t2) and the VAS for general recovery (rs = 0.59). The strength of the relationship was similar to the original version (*r* = 0.68) and translated versions (range: *r* = 0.6 to *r* = 0.63) [1, 5, 7, 8, 19].

In contrast to most previous studies, this study confirmed construct validity by demonstrating the expected gender difference and a significant positive correlation between age and the postoperative QoR-15NL score. These findings have been reported by two and one previous studies, respectively [1, 4]. However, it is unclear why previous studies reported contradictory results regarding the association between gender and age with the postoperative QoR-15 score. It has been established that male gender and older age are associated with increased patient satisfaction with anesthesia in general and higher satisfaction with postoperative recovery after ambulatory surgery and anesthesia [25, 26]. Supposing the latter is also true for inpatient surgery, one hypothesis could be that the patient-perceived satisfaction with their postoperative recovery is positively associated with the QoR-15 score, which would explain this study’s findings.

Future studies should focus on determining the MCID of the QoR-15NL and whether the QoR-15NL is a suitable measure of QoR for Dutch patients undergoing ambulatory surgery under general anesthesia. The QoR-15 could also be validated for patients undergoing surgery under different anesthesia modes (i.e. neuraxial or regional techniques). Additionally, developing an electronic version of the QoR-15, integrated into the electronic patient record, might increase the questionnaire’s clinical feasibility [27]. Finally, future studies may focus on the effect of systematically reporting QoR-15 scores as feedback to anesthesiologists to improve their clinical performance.

## Conclusion

In conclusion, the QoR-15NL scale is a valid, easy-to-use, and reliable outcome assessment tool with high responsiveness for patient-reported quality of recovery after surgery and general anesthesia in the Dutch-speaking population. The measurement properties of the QoR-15NL scale are comparable to both the original version and other translated versions.

## Supplementary Information

**Additional file 1.**

## Data Availability

Following the current study’s Data Management Plan, the datasets generated and/or analyzed are not publicly available but are available from the corresponding author on reasonable request.

## Abbreviations

ANOVA: Analysis of variance
ASA: American Society of Anesthesiologists
CI: Confidence interval
COPD: Chronic obstructive pulmonary disease
COSMIN: COnsensus-based Standards for the selection of health Measurement INstruments
ENT: Ear, nose, or throat
ICC: Intraclass correlation coefficient
ICU: Intensive Care Unit
IQR: Interquartile range
n/a: Not applicable
PACU: Postoperative Anesthesia Care Unit
PONV: Postoperative nausea and vomiting
PROM: Patient-reported outcome measure
QoR: Quality of recovery
QoR-15: 15-item Quality of Recovery scale
QoR-15NL: Dutch 15-item Quality of Recovery scale
r: Pearson’s correlation coefficient
rs: Spearman’s correlation coefficient
SD: Standard deviation
SDC: Smallest detectible change
SRM: Standardised response mean
UMCG: University Medical Center Groningen
VAS: Visual analogue scale
